# Lauric acid as feed additive – An approach to reducing *Campylobacter* spp. in broiler meat

**DOI:** 10.1371/journal.pone.0175693

**Published:** 2017-04-18

**Authors:** Katrin Zeiger, Johanna Popp, André Becker, Julia Hankel, Christian Visscher, Guenter Klein, Diana Meemken

**Affiliations:** 1 Institute for Food Quality and Food Safety, University of Veterinary Medicine Hannover, Foundation, Bischofsholer Damm 15, Hannover, Germany; 2 Institute for Animal Nutrition, University of Veterinary Medicine Hannover, Foundation, Bischofsholer Damm 15, Hannover, Germany; 3 Institute of Agricultural and Nutritional Sciences, Martin Luther University Halle-Wittenberg, Theodor-Lieser-Straße 11, Halle (Saale), Germany; University of Bonn, GERMANY

## Abstract

The increasing prevalence of *Campylobacter* spp. within broiler populations is a major problem for food safety and consumer protection worldwide. *In vitro* studies could already demonstrate that *Campylobacter* spp. are susceptible to lauric acid. The purpose of this study was to examine *in vivo* the influence of lauric acid as a feed additive on slaughter parameters, muscle fatty acid profile, meat quality traits and the reduction of *Campylobacter coli* in inoculated meat of Ross 308 (R308) and Hubbard JA 757 (HJA) broilers in three independent trials (n = 3). Although slaughter parameters did not show any significant differences, the fatty acid profile of both breeds revealed significantly higher lauric acid concentrations (P < 0.0001) in the *Musculus pectoralis superficialis* of treated broilers. Comparing both tested breeds, R308 test broilers had significantly higher lauric acid concentrations than HJA test broilers (P < 0.0001), indicating a higher conversion rate in those animals. The meat quality traits showed no differences in the R308 breed (P > 0.05), but HJA test broilers had higher values for drip loss, electrical conductivity, CIE color values L* and b*, and lower pH values. The inoculation trials of R308 showed that initial bacterial loads of 5.9 log_10_ cfu/g were reduced during six days of storage (4°C) to approximately 4.3 log_10_ cfu/g in the control groups compared to 3.5 log_10_ cfu/g in the treatment groups (P = 0.0295), which could be due to antimicrobial effects of lauric acid within the muscle. This study therefore suggests that lauric acid as a feed additive has the potential to improve food safety by reducing the numbers of *Campylobacter coli* in broiler meat. However, this effect seems to be dependent on the breed determining the feed intake capacity, the fat deposition and therefore the ability to incorporate lauric acid in the muscle.

## Introduction

Nowadays the infection of animals and the contamination of their relating food products with zoonotic pathogens are attracting an increasing amount of consumer attention. While the number of infections with *Salmonella* spp. is continuously decreasing as a result of many successful control programs, the cases of Campylobacteriosis surged to a particularly high level of about 70,000 infections in Germany in 2015 [1]. *Campylobacter* spp., mainly *C*. *jejuni*, *C*. *coli* and *C*. *lari*, were responsible for more than 230,000 cases in the European Union in 2014 [2]. Campylobacteriosis is therefore the most frequently identified foodborne bacterial zoonosis. The main cause of infection originates from poultry being heated inadequately or handled without the necessary caution in the kitchen [3]. As cross-contamination and poor hygiene are believed to be the main reasons for contamination with *Campylobacter* spp. at abattoirs [4, 5], many studies deal with approaches to improving the hygiene management or processing technology in those facilities [6–8]. In this context, the European Food Safety Authority [2] reported that 38.4% of about 6,700 samples collected from abattoirs, processing plants, and retail shops in 2014 were *Campylobacter*-positive. Ways to optimize processing methods are, for example, the use of elevated scalding temperatures [9], or improved chilling methods [10] as these two steps are crucial for reducing the bacterial load. With the aim of reducing the prevalence of *Campylobacter* spp. in broiler flocks, some feeding studies investigated organic acids or medium chain fatty acids (MCFA) as additives in drinking water because of their antimicrobial potential [11–13]. Some studies suggested that there might be an impact of feeding medium chain fatty acids on the reduction of *Campylobacter jejuni* [14, 15]. *In vitro* trials with lauric acid showed that this MCFA has the potential to decrease *Campylobacter jejuni* [16]. However, to our knowledge, there are no *in vivo* studies that have investigated the effects of lauric acid as a feed additive regarding *Campylobacter* spp. prevalence and meat quality. Therefore, the aim of the present study was to investigate the effect of adding palm kernel fatty acid distillates, which contain high amounts of lauric acid, to the diet of two different broiler breeds. The influence on different slaughtering characteristics, meat quality traits as well as the impact on the fatty acid profile of the breast muscle were determined. Regarding antimicrobial effects, an inoculation experiment with *Campylobacter coli* on the *Musculus pectoralis superficialis* was conducted.

## Material and methods

Animal experiments were performed in accordance with the German Animal Welfare Act, and approved by the Ethics Committee for Care and Use of Laboratory Animals of the Lower Saxony State Office for Consumer Protection and Food Safety Lower Saxony LAVES (approval number 33.9-42502-05-15A500).

### Rearing

For this study a fast growing (Ross 308) and a slower growing (Hubbard JA 757) chicken breed with a total number of 180 birds were examined. Each breed consisted of 30 broilers being reared under comparable conditions within three independent trials (n = 3) at the Institute for Animal Nutrition, University of Veterinary Medicine, Foundation, Hannover, Germany. To minimize the influences of potential differences in the rearing conditions the groups were randomly assigned to the different compartments in the barn with a density of 23.3 kg/m^2^ mean metabolic bodyweight at the end of the trial. In the first week, the broilers were fed a common starter feed followed by a commercial grower feed for the following two weeks. After that, the breeds were randomly assigned to two subpopulations (15 broilers, respectively) in order to provide specific diets during the finishing period. The basic finisher feed was a common pellet diet which had been reduced by 2% in its fat content, which was supplemented by 5% of a commercial standard fat (C 12 < 6%) in the case of the control group. In contrast, the test group was finished with a supplement of 5% fat enriched with palm kernel fatty acids including high levels of lauric acid (C 12 = 42–53%) (Table 1). Water and feed were provided ad libitum and controlled twice a day. The feed intake was calculated by subtracting the backweights from the initial weight of food on a daily basis. The project was performed in accordance with the German Animal Welfare Act.

**Table 1:** Fatty acid content of the control diet (5% commercial fat addition) and test diet (5% supplement of fat with high lauric acid content) for the different trials (g/kg dry matter).

trial		1		2		3	
trivial name	number of C-atoms: double bonds	control diet	test diet	control diet	test diet	control diet	test diet
caprylic acid	C8:0	0.00	1.27	0.00	1.22	0.06	1.15
capric acid	C10:0	0.00	1.31	0.00	1.25	0.08	1.15
lauric acid	C12:0	0.49	21.5	0.59	21.6	0.42	19.7
linoleic acid	C18:2	39.6	27.8	43.0	29.5	37.5	25.1

### Slaughtering and sampling

At the age of 41 d (first and third trials) or 42 d (second trial), the broilers were slaughtered. Stunning was performed by a percussive blow to the head and bleeding by ventral neck cutting. Live weight was taken before slaughtering. Immediately after slaughtering, the *Musculi pectorales superficiales* (MPS) were obtained and stored at + 4°C. During cutting, carcasses, thighs and breast muscles were weighed. Twelve left MPS of each breed and trial (in total 72) were minced with a Grindomix (GM 200, Retsch®, Germany), vacuum packed and stored frozen at -20°C until further analysis of the fatty acid profile.

### Meat quality traits

#### pH value measurements

The pH values were measured 24 h post mortem (p. m.) with a portable pH-meter (Portamess® Type 911 pH, Knick, Germany) combined with a glass electrode (InLab 427®, Mettler- Toledo, Urdorf, Switzerland) and a temperature sensor, in the cranial part of the right MPS. Mean values of triplicate measurements were calculated.

#### Electrical Conductivity (EC) measurements

The EC was determined 24 h p. m. in the cranial part of the right MPS with a portable EC meter, equipped with two parallel stainless steel electrodes (LF- Star®, Matthaeus GmbH, Poettmes, Germany). Likewise, mean values of triplicate measurements were calculated.

#### Instrumental color measurements

The CIE L* (lightness), a* (redness), b* (yellowness) color values were measured 24 h p. m. with a colorimeter (Minolta CR 400®, Konica Minolta GmbH, Langenhagen, Germany) on a fresh cut in MPS on the bone-faced side. Mean values of three measuring points per sample were calculated.

#### Drip loss measurements

The drip loss was determined 24 h p. m. After wiping the left PM muscle, they were stored in individual plastic boxes at + 4°C for 48 hours. The drip loss in percentage was calculated as the weight difference between the samples before and after storage.

### Inoculation experiments

#### Sampling

At 24 h p. m., two samples of 25 g, respectively, were obtained from the right MPS. Both samples were stored in plastic boxes until further processing. One colony of a *Campylobacter coli* strain (DSM 4689) was transferred to 10 mL Bolton broth (Oxoid GmbH, Wesel, Germany) and incubated 44±4h under microaerophilic conditions. The final amount of bacteria was approximately 8 log_10_ colony forming units (cfu) per milliliter.

#### Inoculation

For spiking, 0.1 mL per sample of the inoculum was transferred to the surface. Immediately after inoculation one of the two meat samples was conveyed to a stomacher bag with buffered peptone water in a 1:10 dilution for quantitative analysis as a control for the inoculation dose at the outset of the experiment (day 1). After homogenization in a laboratory paddle blender (Stomacher^®^ 400 circulator, Seward Ltd., United Kingdom) at 230 rpm for two minutes, 0.1 mL of the suspension was surface-plated in duplicate on modified Charcoal Cefoperazone Deoxycholate Agar (Oxoid GmbH, Wesel, Germany). The plates were incubated for 48 h under microaerophilic conditions (10% CO2, 5% O2, 85% N2) at 41.5°C. Enumeration was carried out in accordance with ISO 10272-2:2006. The remaining sample was quantitatively analyzed after a storage period of six days at + 4°C in the same manner as mentioned previously (day 7). Samples below the detection limit of 100 cfu/g were included in the calculation as half of the detection limit (50 cfu/g).

### Fatty acid content

The minced meat of the left MPS was freeze-dried in a lyophilization unit (Gamma 1-20, Christ^®^, Osterode, Germany), pulverized in a mixer mill (MM 400, Retsch^®^, Haan, Germany) and analyzed by gaschromatography (GC TRACE 1300, ThermoScientific^®^, Dreieich, Germany; SP-2560 Column, Supelco, Bellefonte, USA; carrier gas: nitrogen) with a modified method according to Lepage and Roy [17]. In brief, a methanol-hexane-tridecanoic-acid mixture was utilized as standard. Subsequently, acetyl chloride was added and the sample was heated, followed by the addition of potassium chloride solution. The measurement was carried out after centrifugation with the superior hexane phase.

### Statistical methods

Statistical analysis was performed by using SAS (Statistic Analyzing Software, version 7.1, Cary, NC, USA). The normal distribution was tested by the Shapiro-Wilk-Test. The significant differences concerning the breeds and fat additions or interactions in between were determined by a two-way analysis of variance (ANOVA). Unless otherwise stated, P-values below 0.05 were considered significant.

## Results and discussion

### Slaughtering characteristics

Both R308 and HJA showed no differences in the slaughtering characteristics between the control and the test group (P > 0.05) (Table 2). These results are in accordance with Zeitz, Fennhoff [18], who also found that feeding lauric acid had no negative impact on performance characteristics. In contrast, van der Hoeven-Hangoor, van der Vossen [19] found that it is possible to enhance broiler performance by adding medium chain fatty acids (capric and lauric acid) to the diet. However, when comparing both breeds, R308 had significantly higher values for the live, carcass, thigh and breast muscle weights (P < 0.001) (Table 2). This was due to the higher feed intake capacity of R308 (56017 g ± 4216 for the control and 54267 g ± 3773 for the test group) compared to the feed intake capacity of HJA (40017 g ± 2705 and 40197 g ± 5000 for control and test group, respectively) (Table 3). These findings are in line with Acar, Moran [20] and Mehaffey, Pradhan [21], who also found that different breeds have altering performance characteristics.

**Table 2:** Mean values (±standard deviation) of the slaughter characteristics of Ross 308 (R308) and Hubbard JA 757 (HJA) broilers, fed with 5% commercial fat addition (control group) and broilers fed with 5% supplement of fat with high lauric acid content (test group) (n = 3).

	R308						HJA					
weight (g)	control group			test group			control group			test group		
live	2877^a^	±	499	2794 ^a^	±	328	1821 ^b^	±	289	1897 ^b^	±	303
carcass	2316 ^a^	±	397	2292 ^a^	±	246	1482 ^b^	±	274	1527 ^b^	±	219
thigh	524 ^a^	±	98	509 ^a^	±	88	344 ^b^	±	66	358 ^b^	±	67
breast muscle	565 ^a^	±	126	564 ^a^	±	81	271 ^b^	±	63	279 ^b^	±	67

^a,b^ values within a line followed by different letters differ significantly (P<0.05).

**Table 3:** Mean values (± standard deviation) of the total feed intake of Ross 308 (R308) and Hubbard JA 757 (HJA) broilers fed with 5% commercial fat addition (control group) and broilers fed with 5% supplement of fat with high lauric acid content (test group) (n = 3).

	R308			HJA		
control group	56017 ^a^	±	4216	40017 ^b^	±	2705
test group	54267 ^a^	±	3773	40197 ^b^	±	5000

^a,b^ values within a line followed by different letters differ significantly (P<0.05)

### Meat quality traits

There were no differences between the test and the control group of the R308 breed for the meat quality traits drip loss, EC, pH, L*, a*, and b* (P > 0.05) (Table 4). Concerning the HJA breed, drip loss and EC (1.30 ± 0,90% and 9.40 ± 3,01 mS, respectively) of the test group were higher compared to the control group (1.03 ± 0,28%, 7.38 ± 2,8 mS) (P < 0.05), while the pH values of the test broilers was lower than the pH values of the control group (P < 0.05) (Table 4). Several studies [22–24] postulated that drip loss is positively correlated to EC and negatively correlated to pH, which is in line with the present observations. The water-holding capacity is influenced by pH because of the protein denaturation, which occurs p. m. as a consequence of the release of H^+^-ions [25] and because of the physiologic, and enzymatic degradation of cell membranes [26]. Correspondingly, EC is also related to the relocating of ions and cell fluids by the degradation of cell membranes [22]. The observation of differences between the diet groups in the HJA breed was unexpected because R308 had higher feed intake and consequently higher intake of lauric acid than HJA. Thus, the assumption was that, if there were significant differences they would have been found in the R308 breed. The addition of lauric acid appears to have only a significant influence on the pH value and therefore on the other meat quality parameters in the HJA breed. Reasons for this difference in the lauric acid effect between the two breeds are not known and further investigations are necessary.

**Table 4:** Mean values (±standard deviation) of the meat quality traits of Ross 308 (R308) and Hubbard JA 757 (HJA) broilers fed with 5% commercial fat addition (control group) and broilers fed with 5% supplement of fat with high lauric acid content (test group) (n = 3).

	R308						HJA					
	control group			test group			control group			test group		
drip loss (%)	1.54^a^	±	0.56	1.24^a^	±	0,69	1.03^b^	±	0,28	1.30^a^	±	0.90
EC (mS^1^/cm)	6.63^b^	±	1.99	7.42^b^	±	2,47	7.38^b^	±	2,80	9.40^a^	±	3.01
pH	5.75^a^	±	0.11	5.73^a^	±	0,11	5.70^a^	±	0,17	5.61^b^	±	0.10
L*	53.47^a^	±	2.64	52.52^a^^b^	±	2,82	51.39^b^	±	3,68	52.86^a^	±	3.58
a*	3.70^a^	±	1.82	3.07^a^^b^	±	1,68	3.16^a^^b^	±	1,33	3.01^b^	±	1.31
b*	4.97^b^	±	1.38	5.29^b^	±	1,55	5.42^b^	±	1,71	6.25^a^	±	1.68

^a,b^ values within a line followed by different letters differ significantly (P<0.05).

^1^ millisiemens

The MPS of the test group had higher L* and b* values than the controls (P < 0.05), but there was no difference observed for a* values (P > 0.05) (Table 4). Otto and Roehe [24] found that paler meat is related to higher drip loss, which is in keeping with our results. This lighter color is due to the fact that higher drip loss and the protein denaturation alters the light scattering on the surface of meat.

### Fatty acid profile

The analysis of the fatty acid profile clearly showed higher contents of lauric acid (C 12) within the breast muscles of the test groups than both control groups (P < 0.0001) (Table 5). This is most probably the result of the test diet containing more than forty times higher concentrations (20.9 g/kg feed dry matter) of lauric acid than the control diet (0.5 g/kg feed dry matter) which was incorporated in the muscle matrix. Our results are consistent with those reported by Roth and Ristic [27] who also showed that it is possible to alter the fatty acid profile of broilers by providing a particular diet of rapeseed oil.

**Table 5:** Mean values (± standard deviation) of the fatty acid profile (left MPS) of Ross 308 (R308) and Hubbard JA 757 (HJA) broilers fed with 5% commercial fat addition (control group) and broilers fed with 5% supplement of fat with high lauric acid content (test group) (n = 3).

	R308						HJA					
g/kg dry matter	control group			test group			control group			test group		
C8	0.03 ^c^	±	0.02	0.07 ^a^	±	0.02	0.03 ^c^	±	0.01	0.05 ^b^	±	0.02
C10	0.02 ^c^	±	0.01	0.20 ^a^	±	0.06	0.02 ^c^	±	0.01	0.11 ^b^	±	0.08
C11	0.03 ^c^	±	0.01	0.05 ^a^	±	0.01	0.03 ^c^	±	0.01	0.04 ^b^	±	0.01
C12	0.25 ^c^	±	0.12	6.35 ^a^	±	1.89	0.18 ^c^	±	0.07	3.41 ^b^	±	2.27
C14	0.49 ^c^	±	0.17	4.47 ^a^	±	1.38	0.25 ^c^	±	0.11	2.43 ^b^	±	1.47
C16_0	19.26 ^a^	±	6.14	19.30 ^a^	±	5.78	9.96 ^b^	±	4.10	12.01 ^b^	±	5.81
C16_1	1.90 ^a^	±	0.84	2.39 ^a^	±	0.85	0.75 ^c^	±	0.56	1.34 ^b^	±	0.93
C17_0	0.12 ^a^	±	0.03	0.10 ^a^^b^	±	0.03	0.06 ^b^^c^	±	0.02	0.08 ^b^	±	0.07
C18_0	6.38 ^a^	±	1.42	6.39 ^a^	±	1.83	3.95 ^b^	±	0.87	4.21 ^b^	±	1.11
C18_1n9t	0.52 ^a^	±	0.19	0.32 ^b^	±	0.16	0.28 ^b^^c^	±	0.12	0.21 ^c^	±	0.14
C18_1n9c	26.14 ^a^	±	9.10	24.56 ^a^	±	7.58	12.08 ^b^	±	6.25	14.59 ^b^	±	7.80
C18_2n6c	18.47 ^a^	±	6.38	14.80 ^b^	±	4.66	9.56 ^c^	±	3.81	9.91 ^c^	±	4.45
C18_3n3	0.77 ^a^	±	0.32	0.84 ^a^	±	0.32	0.34 ^b^	±	0.19	0.50 ^b^	±	0.31
C20_2	0.39 ^a^	±	0.07	0.35 ^a^	±	0.09	0.26 ^b^	±	0.05	0.25 ^b^	±	0.06
C20_3n6	0.27	±	0.08	0.28	±	0.12	0.24	±	0.03	0.25	±	0.03
C20_4n6	1.84	±	0.46	1.67	±	0.57	1.85	±	0.38	1.58	±	0.19
C22_6n3	0.11 ^b^	±	0.04	0.12 ^b^	±	0.04	0.16 ^a^	±	0.06	0.17 ^a^	±	0.04

^a,b,c^ values within a line followed by different letters differ significantly (P<0.05)

Regarding the potential of utilizing lauric acid, R308 broilers seem to have a higher conversion rate as they had two-fold higher levels of absolute lauric acid content in their MPS compared to the HJA breed (P < 0.0001), whereas no difference between the control groups occurred (P = 0.8751) (Table 5). This could be a result of the higher feed intake, as described above, and thereby related higher intake of lauric acid of R308, being a fast growing breed with exalted feed intake performance compared to HJA. In addition, the R308 test group had a higher total fat content (82.27 g/kg) compared to the HJA test group (51.14 g/kg) and therefore higher absolute lauric acid levels (7.7% and 6.7% for R308 and HJA, respectively) (Table 5). This leads to the assumption that lauric acid could be more effective in breeds that are inclined to have higher fat depositing abilities because the higher the total fat content in the muscle, the higher the absolute lauric acid levels could be.

### Inoculation

**Fig 1:**
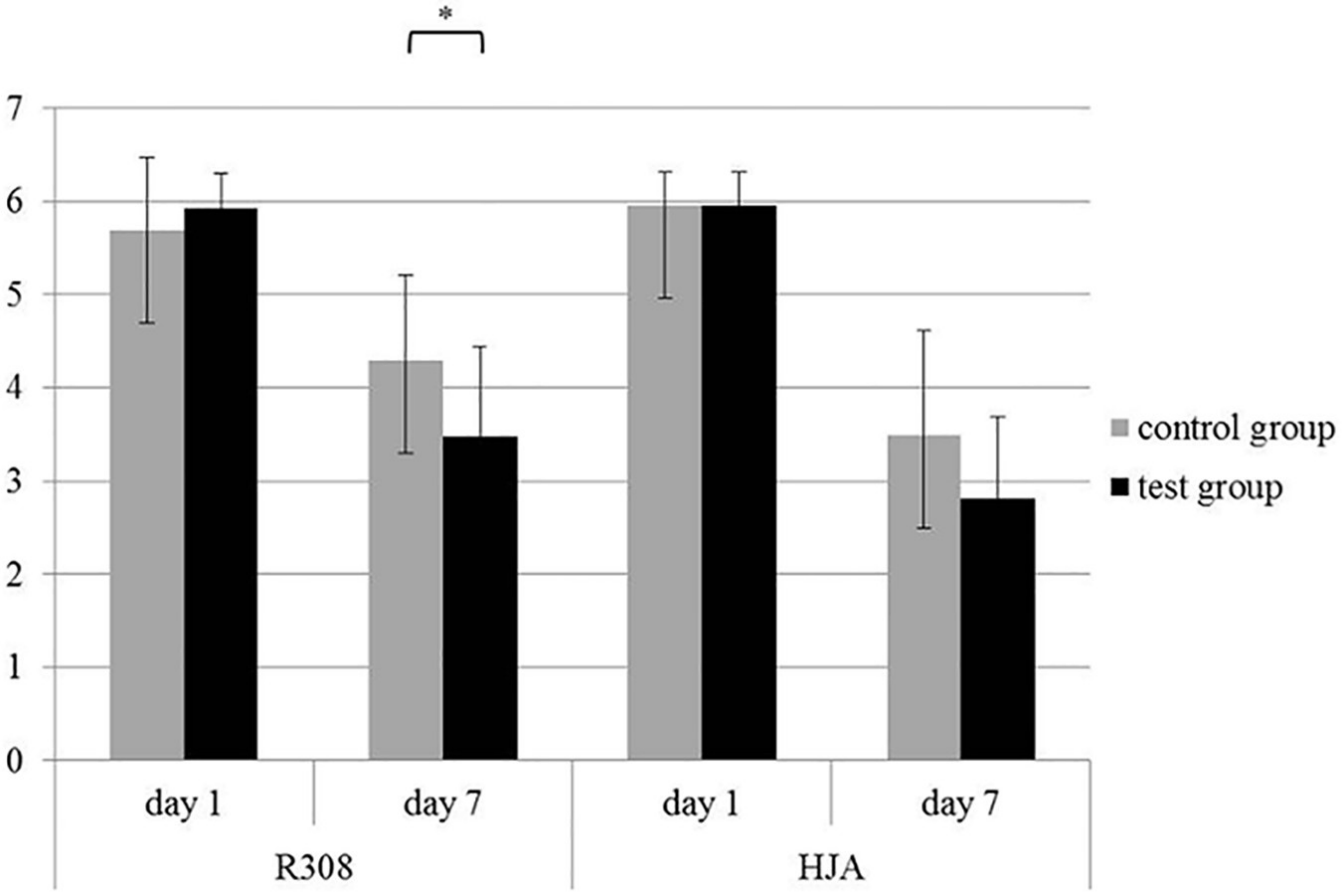
Inoculations experiment: Bacterial counts (mean values ± SD) of *Campylobacter* spp. at the beginning (day 1) and end (day 7) in log_10_ cfu/g of Ross 308 (R308) and Hubbard JA 757 (HJA) broilers fed with 5% commercial fat addition (control group) and broilers fed with 5% supplement of fat with high lauric acid content (test group). The asterisk (*) marks significant differences (P < 0.05). There were no significant differences between day 1 and 7 and within the feeding groups (P > 0.05) (n = 3).

As expected the starting values of day 1 did not vary (P > 0.05) between the test and control group, with an average value of approximately 6 log_10_ cfu/g in both breeds (Fig 1). This bacterial count of day 1 served as a control for the adequate procedure of this inoculation experiment. The value was approximately 1 log_10_ higher than average counts that are usually found in commercial broiler slaughterhouses on naturally contaminated carcasses of *Campylobacter* positive flocks [28]. After six days of storage (day 7), the microbial loads of *C*. *coli* of the R308 breed control group dropped from approximately 5.7 log_10_ to 4.3 log_10_ cfu/g, while the test group showed a higher reduction of initially 5.9 log_10_ to 3.5 log_10_ cfu/g (Fig 1). As a result, the bacterial loads of the test groups were significantly lower after storage time (P = 0.0295). As for the HJA breed, there was also a clear decrease from initially 6 log_10_ cfu/g (both control and test groups) to levels of 3.5 log_10_ cfu/g (control) and 2.8 log_10_ cfu/g (test) (Fig 1). However, in this case, only a tendency of lower values in the test group after storage time was distinguishable (P = 0.0685). The sensitivity of *Campylobacter* spp. towards their growth conditions explains the drop in germ counts during refrigerated storage. This observation is in accordance with investigations by Chan, Tran [29] and Oyarzabal, Oscar [30] who showed a reduction of *Campylobacter* spp. at low temperatures (4°C) as well. Our study shows that it is possible to reduce these bacteria on the meat by approximately 1 log_10_ cfu/g by adding lauric acid to the diet. This could be attributed to the higher content of lauric acid in the breast muscle meat. These results are in line with the *in vitro* described ability of lauric acid to reduce *Campylobacter jejuni* [16]. The exact antimicrobial mechanism of lauric acid cannot be clarified in this study. This medium chain fatty acid is assumed to have the ability to destabilize the cell-membrane, followed by cell-degeneration. For *Salmonella* spp. and *Escherichia coli* this was visible in disorganized cytoplasm, while *Clostridium perfringens* showed a detachment between the inner side and outer surface of the cell-membrane. A change in the permeability of K^+^-ions was not detectable [31, 32]. Similar findings were made concerning *Campylobacter jejuni* by Molatova, Skrivanova [16], who also revealed disorganization of cytoplasm of *C*. *jejuni* cells after treatment with lauric acid.

## Conclusion

We therefore conclude that lauric acid as a feed additive could possibly improve the food safety of broiler meat because of its ability to reduce the bacterial load with *Campylobacter coli*. However, broiler breed specific characteristics have to be taken into account as there were considerable differences between the breeds tested in the present study. Further research has to investigate these influences and clarify whether these observations are transferable to other food-borne pathogens like *Salmonella* spp. or *Listeria* spp. which are able to grow during refrigerated storage [33].
